# Ocular motor cranial nerve palsy and increased risk of stroke in the general population

**DOI:** 10.1371/journal.pone.0205428

**Published:** 2018-10-15

**Authors:** Sang Jun Park, Hee Kyung Yang, Seong Jun Byun, Kyu Hyung Park, Jeong-Min Hwang

**Affiliations:** Department of Ophthalmology, Seoul National University College of Medicine, Seoul National University Bundang Hospital, Seongnam, Republic of Korea; University of L’Aquila, ITALY

## Abstract

**Purpose:**

To determine whether ocular motor cranial nerve (CN) palsy raises the risk of subsequent stroke in the general population.

**Methods:**

We investigated the association between ocular motor CN palsy and occurrence of stroke using the National Health Insurance Service-National Sample Cohort database from 2002 to 2013. We included individuals aged ≥ 20 years on January 1^st^, 2004, and excluded those having any paralytic strabismus, disorders in binocular movement, diplopia and any cerebrovascular diseases before entering the cohort. Incident ocular motor CN palsy was identified by diagnostic codes for third, fourth, and sixth nerve palsies. To determine the effect of incident ocular motor CN palsy on the occurrence of subsequent stroke, we used time-varying covariate Cox regression models. Model 1 included only incident third, fourth, and sixth nerve palsies as a time-varying covariate. Model 2 included Model 1 and defined demographic information. Model 3 included Model 2, comorbidity, co-medication, and the Charlson index score.

**Results:**

Among 727,689 individuals in the cohort, 1,633 patients developed ocular motor CN palsy and 17,657 patients suffered stroke. Cox regression models showed that development of ocular motor CN palsy was associated with an increased risk of subsequent stroke (hazard ratio [HR] = 4.65; 95% confidence intervals [CIs], 3.74–5.80 in Model 1), and the results were consistent even after adjusting for demographic factors and confounders in Model 2 and 3. Men, older age, and individuals not living in Seoul/Incheon area were associated with an increased risk of stroke, while individuals with higher income were associated with decreased risk of stroke in both Model 2 and 3. Sensitivity analyses using propensity score-based matching produced similar results in all three Models (HR = 1.95; 95% CI, 1.55–2.46 in Model 1, HR = 1.91; 95% CI, 1.52–2.41 in Model 2, and HR = 1.63; 95% CI, 1.29–2.06 in Model 3).

**Conclusions:**

The occurrence of ocular motor CN palsy is a significant risk factor of subsequent stroke even after adjusting for demographic factors and confounders in the general population. Physicians may need to educate patients with ocular motor CN palsy regarding the higher risk of future stroke.

## Introduction

Stroke is one of the global leading causes of disability, and its prevalence has increased dramatically.[1] Risk factor modification and identification of predictive factors are essential for prevention of stroke.[1–3]

Meanwhile, isolated third, fourth, and sixth nerve palsies are commonly associated with microvascular ischemic conditions such as diabetes mellitus, hyperlipidemia, and hypertension.[4] Stroke and ocular motor CN palsy are both commonly associated with arteriosclerotic conditions such as diabetes mellitus, hyperlipidemia, and hypertension.[5] There have been two reports suggesting that ocular motor cranial nerve (CN) palsy may be an unrecognized risk factor of stroke.[5, 6] However, whether the event of isolated ocular motor CN palsy is an independent risk factor of stroke or not is still unclear. In fact, the compound impact of ocular motor CN palsy on the risk of stroke has not been investigated in a very large scale.

Herein, in this study, we performed a nationwide cohort study including a whole large population-based database of individuals aged 20 years or older up to 7,008,001 person-years to determine the risk of stroke in those who developed third, fourth, and sixth nerve palsies, after adjusting for demographic factors, covariates, and the use of co-medications.

## Methods

### Study population

We used the National Health Insurance Service (NHIS)-National Sample Cohort (NSC) database for this study. The NHIS is a single, compulsory medical insurance program in South Korea which started in 1977 and achieved universal coverage by 1989.[7–9] Therefore, the NHIS contains all information regarding healthcare utilization in Korea. The NHIS-NSC database consists of a random sample of 1,025,340 Korean residents, equivalent to approximately 2.2% of the Korean population in 2002. The database contains 12 years of claims from 2002 to 2013 for diagnoses, procedures, prescription records, demographic information, direct medical costs, and mortality without any duplications or omissions.[9]

The diagnosis was coded according to the Korean Classification of Disease, 6th edition (a version of the International Classification of Diseases, 10th edition, adapted for the Korean healthcare system). The validation study showed an overall positive predictive value of the diagnosis as 83.4% by comparing the diagnoses between the database and the patients’ medical record.[10] Detailed information regarding the NHIS and database has been reported elsewhere.[2, 3, 9, 11–16] The database is open to any researcher whose study protocols are approved by the official review committee. All patient records were de-identified and analyzed anonymously.

### Cohort definition

Using the NHIS-NSC database, we defined the fixed cohort to investigate the association between ocular motor CN palsy and subsequent development of stroke, which started on January 1^st^, 2004, and ended on December 31^st^, 2013. We included individuals aged 20 years or older when entering the cohort on January 1^st^, 2004, and excluded those having any paralytic strabismus (H49), disorders in binocular movement (H51), diplopia (H53.2) and any cerebrovascular diseases (I60–I68, G45, and G46) before entering the cohort. Lastly, of 1,025,340 Korean residents in the NHIS-NSC database, a total of 727,689 individuals entered the cohort.

### Definition of ocular motor cranial nerve palsy and confounders

We identified the incident of ocular motor CN palsy by the use of diagnostic codes for third nerve palsy (H49.0), fourth nerve palsy (H49.1), and sixth nerve palsy (H49.2) among the defined cohort. For statistical analysis, we defined age, sex, residential region, household income, comorbidity, and co-medication as possible confounders of the association between third, fourth, and sixth nerve palsies and stroke.

We defined the presence of comorbidities and the use of co-medications according to previous diagnoses and previous prescriptions up to two-years before entering the index date. We calculated the modified Charlson comorbidity index by the use of previous diagnosis within one year before the index date.[8, 17] Defined comorbidities were hypertension, diabetes, ischemic heart disease, atrial fibrillation, congestive heart failure, peripheral artery disease, cancer, tuberculosis, chronic kidney disease and dyslipidemia. Defined information on co-medication included the use of low-dose acetylsalicylic acid, platelet aggregation inhibitors, warfarin, heparin, anti-thrombotic agents, anti-hypertensive agents, oral hypoglycemic agents, and insulin.

### Statistical analysis

We defined the outcome as the time to first hospital admission with stroke (I60, I61, I62, I63, and I64) after entering the cohort. We defined censoring only when cases died or at the end of follow-up (December 31^st^, 2013). To determine the effect of incident third, fourth, and sixth nerve palsies on the development of stroke, we used time-varying covariate Cox regression models; Model 1 included only incident third, fourth, and sixth nerve palsies as a time-varying covariate. Model 2 included Model 1 and defined demographic information, and Model 3 included Model 2, comorbidity, co-medication, and the Charlson index score. We plotted Kaplan-Meier curves for stroke according to the presence of ocular motor CN palsy which was estimated with the time-varying covariate Cox regression model of Model 1.

Subgroup analysis #1 was performed to investigate the individual effect of each type of ocular motor CN palsy (third, fourth, and sixth nerve palsy) on the development of stroke in the similar way as stated above.

Subgroup analysis #2 was performed according to the type of stroke, after defining the outcomes as the time to first hospital admission due to hemorrhagic stroke (I60-I62) or ischemic stroke (I63).

We performed a sensitivity analysis by the use of propensity score-based matching in the defined cohort. We estimated propensity scores for individuals in the cohort on the basis of cohort entering date without regard to outcome. Multiple logistic regression analysis was performed using age, sex, residential region, household income, comorbidity, co-medication, and Charlson index category. We assessed the model discrimination with C-statistics. Finally, we identified 1,633 patients suffering ocular motor CN palsy throughout the study period. We matched 10 controls among individuals without any ocular motor CN palsy to each identified CN palsy patient by the use of optimal matching with estimated propensity scores. We compared baseline characteristics between patients suffering ocular motor CN palsy and their matched controls in reference to the cohort entering date. We defined the same outcome stated above which was the time to first hospital admission with stroke (I60, I61, I62, I63, and I64) after entering the cohort. Cox regression models (Model 1, 2, and 3), Kaplan-Meier curves, and subgroup analysis #1 and #2 were analyzed in the sensitivity analysis as stated above.

We used SAS software version 9.3 (SAS Inc., Cary, NC) and R programming version 3.1.0 (The R Foundation for Statistical Computing, Vienna, Austria, http://www.R-project.org) for all analyses. *P* values less than 0.05 were considered statistically significant. This study was approved by the institutional review board (IRB) of the Seoul National University Bundang Hospital. The study complied with the guidelines of the Declaration of Helsinki.

## Results

**Fig 1:**
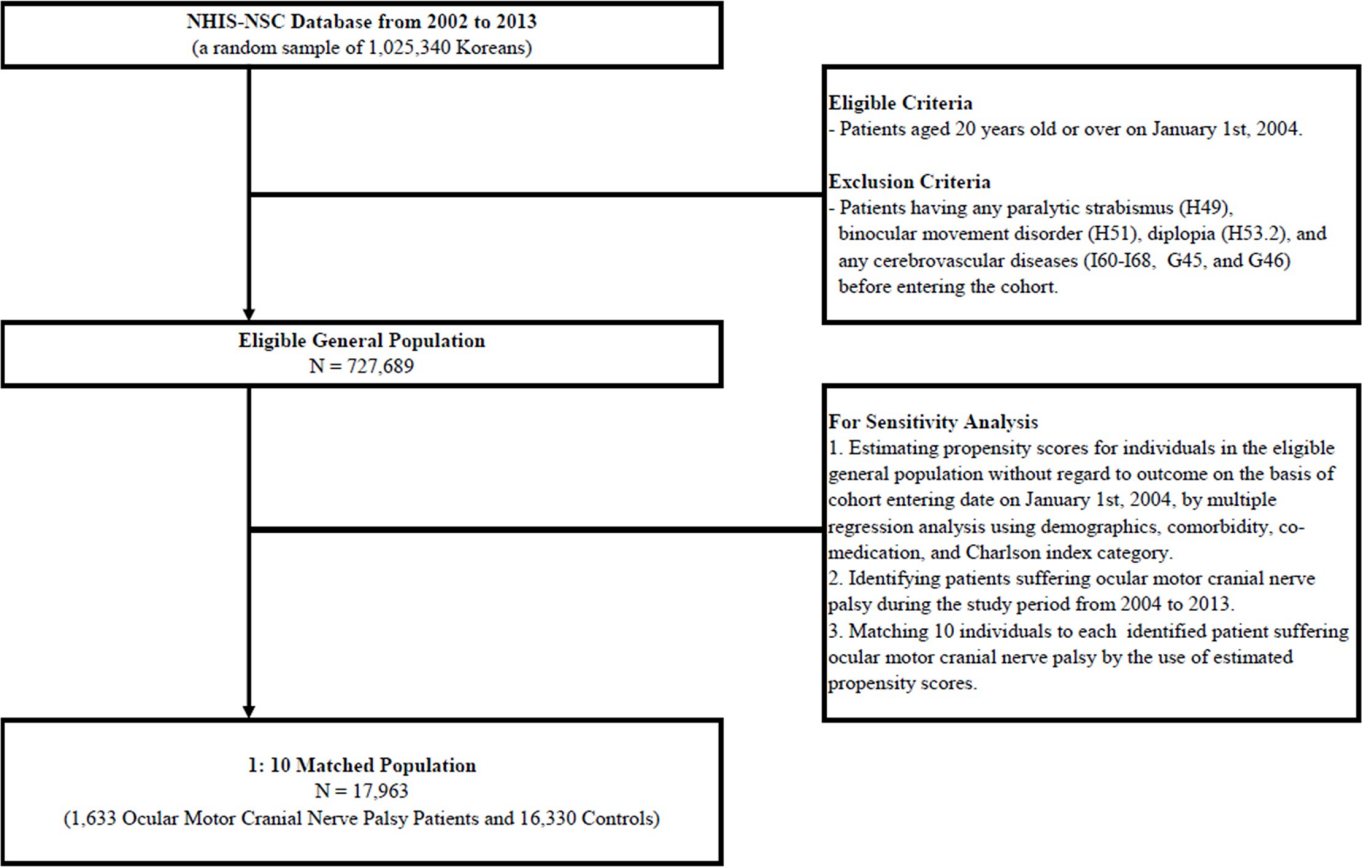
Flow chart according to eligible criteria of main analyses and sensitivity analyses.

Finally, 727,689 individuals (370,269 women, 50.9%) in the cohort were examined and 7,008,001 person-years were included in the analysis. Person-years were derived from the sum of years of observation per person during follow-up until death or end of follow-up. **Fig 1** shows the flow chart along with the eligible criteria. Among them, 1,633 patients developed ocular motor cranial nerve palsy and 17,657 patients suffered stroke. **Table 1** summaries the characteristics of individuals in the cohort. **Table 2** shows an increased risk of stroke after development of third, fourth, and sixth nerve palsies (hazard ratio [HR] = 4.65; 95% confidence intervals [CIs], 3.74–5.80 in Model 1) even after adjusting for demographics (HR = 2.19; 95% CI, 1.76–2.73 in Model 2) and confounders (HR = 1.88; 95% CI, 1.51–2.34 in Model 3) by time-varying cox regression models.

**Table 1:** Demographics and characteristics of eligible population included in the analysis and those with incident cranial nerve palsy and incident stroke.

	Eligible Population	Cranial Nerve Palsy	Stroke
Number	727,689 (100%)	1,633 (100%)	17,657 (100%)
**Demographics**			
Sex			
Women	370,269 (50.9%)	728 (44.6%)	8,538 (48.4%)
Men	357,420 (49.1%)	905 (55.4%)	9,119 (51.6%)
Age group at diagnosis (years)			
20–39	339,306 (46.6%)	232 (14.2%)	1,177 (6.7%)
40–59	268,953 (37.0%)	765 (46.8%)	5,477 (31.0%)
60+	119,430 (16.4%)	636 (38.9%)	11,003 (62.3%)
Residential Area			
Seoul and Incheon	194,793 (26.8%)	430 (26.3%)	3,629 (20.6%)
Gyeonggi and Gangwon	174,442 (24.0%)	372 (22.8%)	3,849 (21.8%)
Busan, Daegu, Ulsan, and Gyeongsang	198,472 (27.3%)	442 (27.1%)	5,502 (31.2%)
Daejeon, Sejong, and Chungcheong	72,926 (10.0%)	176 (10.8%)	2,079 (11.8%)
Gwangju, Jeola, and Jeju	87,056 (12.0%)	213 (13.0%)	2,598 (14.7%)
House Income			
Low income	236,254 (32.5%)	524 (32.1%)	6,359 (36.0%)
Middle income	303,354 (41.7%)	631 (38.6%)	6,460 (36.6%)
High income	188,081 (25.8%)	478 (29.3%)	4,838 (27.4%)
**Comorbidity**			
Hypertension	80,830 (11.1%)	505 (30.9%)	6,551 (37.1%)
Diabetes	49,972 (6.9%)	490 (30.0%)	3,053 (17.3%)
Ischemic Heart Disease	24,178 (3.3%)	144 (8.8%)	1,759 (10.0%)
Congestive Heart Failure	9,480 (1.3%)	52 (3.2%)	959 (5.4%)
Cancer	16,903 (2.3%)	76 (4.7%)	752 (4.3%)
Tuberculosis	8,835 (1.2%)	26 (1.6%)	361 (2.0%)
Peripheral Arterial Disease	13,405 (1.8%)	80 (4.9%)	984 (5.6%)
Atrial Fibrillation	2,916 (0.4%)	17 (1.0%)	339 (1.9%)
Chronic Kidney Disease	1,472 (0.2%)	13 (0.8%)	126 (0.7%)
Dyslipidemia	49,835 (6.8%)	304 (18.6%)	2,449 (13.9%)
**Co-medication**			
Anti-coagulant agents	603 (0.1%)	7 (0.4%)	80 (0.5%)
Anti-hypertensive agents	33,360 (4.6%)	244 (14.9%)	2,822 (16.0%)
Anti-platelet agents	4,558 (0.6%)	37 (2.3%)	466 (2.6%)
Hypoglycemic agents	16 (0.0%)	1 (0.1%)	3 (0.0%)

**Table 2:** Results of time-varying cox regression models for stroke in the eligible population.

	Model 1	Model 2	Model 3
**Time-varying Covariate**			
Cranial Nerve Palsy	4.65 (3.73–5.79)	2.21 (1.77–2.75)	1.70 (1.37–2.12)
**Demographics**			
Sex			
Men	N/A	1 (reference)	1 (reference)
Women	N/A	0.73 (0.71–0.75)	0.72 (0.70–0.74)
Age Group (years)			
20–39	N/A	1 (reference)	1 (reference)
40–59	N/A	6.01 (5.64–6.40)	4.26 (3.99–4.54)
60+	N/A	31.85 (29.98–33.83)	15.11 (14.15–16.13)
Residence			
Seoul and Incheon	N/A	1 (reference)	1 (reference)
Gyeonggi and Gangwon	N/A	1.16 (1.10–1.21)	1.14 (1.09–1.19)
Busan, Daegu, Ulsan, and Gyeongsang	N/A	1.32 (1.26–1.38)	1.38 (1.32–1.44)
Daejeon, Sejong, and Chungcheong	N/A	1.26 (1.19–1.32)	1.26 (1.19–1.33)
Gwangju, Jeola, and Jeju	N/A	1.25 (1.19–1.31)	1.27 (1.21–1.34)
House Income			
Low income	N/A	1 (reference)	1 (reference)
Middle income	N/A	0.92 (0.89–0.96)	0.89 (0.86–0.92)
High income	N/A	0.88 (0.85–0.91)	0.83 (0.80–0.86)
**Covariates**			
Hypertension	N/A	N/A	2.52 (2.42–2.62)
Diabetes	N/A	N/A	1.29 (1.24–1.33)
Ischemic Heart Disease	N/A	N/A	1.11 (1.07–1.16)
Congestive Heart Failure	N/A	N/A	1.29 (1.23–1.35)
Cancer	N/A	N/A	1.00 (0.95–1.04)
Tuberculosis	N/A	N/A	1.02 (0.95–1.08)
Peripheral Arterial Disease	N/A	N/A	1.15 (1.11–1.20)
Atrial Fibrillation	N/A	N/A	1.79 (1.68–1.91)
Chronic Kidney Disease	N/A	N/A	1.55 (1.42–1.70)
Dyslipidemia	N/A	N/A	0.93 (0.89–0.96)
**Co-medications**			
Anti-coagulant agents	N/A	N/A	1.11 (0.89–1.39)
Anti-hypertensive agents	N/A	N/A	0.91 (0.87–0.95)
Anti-platelet agents	N/A	N/A	1.03 (0.93–1.14)
Hypoglycemic agents	N/A	N/A	2.57 (0.83–7.96)

Model 3 included Charlson comorbidity index in the analysis

**Fig 2:**
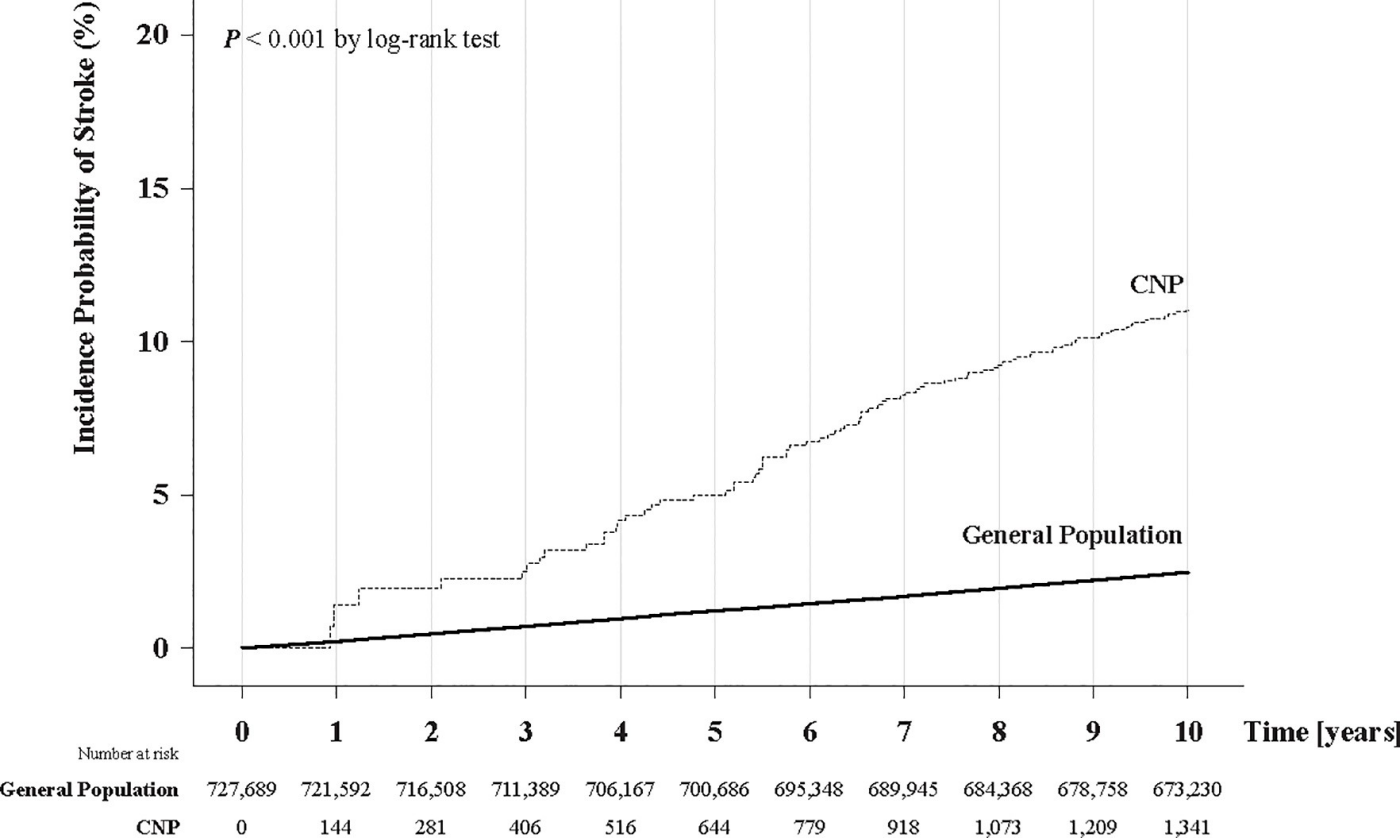
10-year incident rate of stroke according to occurrence of ocular motor cranial nerve palsy. Kaplan-Meier curves show the time interval from study enrollment to the incidence of stroke. Occurrence of ocular motor cranial nerve palsy increased the probability of incident stroke when estimated with time-varying covariate cox regression analysis (*P* < 0.001, log-rank test).

Men, older age group, and individuals not living in Seoul/Incheon area were associated with an increased risk of stroke, while individuals with higher income were associated with decreased risk of ischemic stroke in both Model 2 and 3. Hypertension, diabetes, ischemic heart disease, congestive heart failure, chronic kidney diseases, peripheral arterial disease, and atrial fibrillation were associated with the development of stroke, while dyslipidemia and the use of anti-hypertensive agents were associated with a decreased risk of stroke in Model 3 (**Table 2**). Fig 2 plots the Kaplan-Meier curves for the incident probability of stroke in the cohort according to development of third, fourth, and sixth cranial nerve palsies; individuals who suffered ocular motor CN palsy showed a higher incident probability of stroke (p < 0.001, log-rank test).

Subgroup analysis according to the type of ocular motor nerve palsy (subgroup analysis #1) showed consistent results for third (**Table A in S1 File**) and sixth nerve palsy (**Table C in S1 File**), while fourth nerve palsy (**Table B in S1 File**) was not associated with an increased risk of subsequent stroke in Model 3.

Subgroup analysis according to the type of stroke (subgroup analysis #2) showed consistent results for ischemic stroke (**Table D in S1 File**). However, in hemorrhagic stroke (**Table E in S1 File**), only Model 1 was associated with an increased risk of subsequent stroke, which was no longer significant after adjusting for demographic factors and confounders in Model 2 and 3.

Results of subgroup analysis #1 and #2 are summarized in **Table 3** and the detailed results are presented in **Tables A, B, C, D, and E in S1 File**, respectively.

**Table 3:** Results of time-varying cox regression models for the main analyses and subgroup analyses in both the eligible population and propensity score-based matched population.

	Main Analysis	Subgroup Analysis				
		Subgroup Analysis #1			Subgroup Analysis #2	
Eligible Population	General Population (N = 727,689)					
Variable of Interest	Any CNPs	3^rd^ CNP	4^th^ CNP	6^th^ CNP	Any CNPs	Any CNPs
Outcome	Any Stroke	Any Stroke	Any Stroke	Any Stroke	Ischemic Stroke	Hemorrhagic Stroke
Cox Regression Models						
Model 1, OR (95% CI)	4.65 (3.73–5.79)	5.65 (3.46–9.23)	3.93 (2.04–7.55)	4.58 (2.76–7.61)	5.31 (4.20–6.72)	2.78 (1.64–4.69)
Model 2, OR (95% CI)	2.21 (1.77–2.75)	2.67 (1.63–4.36)	1.99 (1.04–3.83)	2.40 (1.44–3.98)	2.41 (1.90–3.05)	1.53 (0.90–2.58)
Model 3, OR (95% CI)	1.70 (1.37–2.12)	2.15 (1.32–3.51)	1.72 (0.90–3.31)	1.83 (1.10–3.04)	1.81 (1.43–2.29)	1.22 (0.72–2.06)
**Sensitivity Analyses**						
Eligible Population	Propensity Score-based Matched Population (N = 17,963 consisting of 1,633 CNP patients and 16,330 controls)					
Variable of Interest	Any CNPs	3^rd^ CNP	4^th^ CNP	6^th^ CNP	Any CNPs	Any CNPs
Outcome	Any Stroke	Any Stroke	Any Stroke	Any Stroke	Ischemic Stroke	Hemorrhagic Stroke
Cox Regression Models						
Model 1, OR (95% CI)	1.95 (1.55–2.46)	2.13 (1.27–3.57)	1.96 (0.99–3.92)	1.99 (1.17–3.39)	2.10 (1.63–2.69)	1.53 (0.88–2.64)
Model 2, OR (95% CI)	1.91 (1.52–2.41)	2.20 (1.31–3.69)	1.86 (0.93–3.72)	1.98 (1.16–3.38)	2.06 (1.60–2.64)	1.49 (0.86–2.59)
Model 3, OR (95% CI)	1.63 (1.29–2.06)	2.03 (1.20–3.43)	1.55 (0.77–3.11)	1.61 (0.94–2.77)	1.71 (1.33–2.20)	1.32 (0.75–2.29)

CN, Cranial Nerve; CNP, Cranial Nerve Palsy; OR, odds ratio; CI, confidence interval, N, number

Sensitivity analysis included 1,633 patients suffering ocular motor CN palsy and 16,330 controls by the use of estimated propensity scores in reference to the cohort entering date on January 1^st^, 2004. We used a standardized mean difference to compare baseline characteristics between patients with ocular motor CN palsy and their matched controls in **Table F in S1 File**. Sensitivity analysis revealed the robustness of the original analysis; sensitivity analysis in all three Models produced consistent results (HR = 1.95; 95% CI, 1.55–2.46 in Model 1, HR = 1.91; 95% CI, 1.52–2.41 in Model 2, and HR = 1.63; 95% CI, 1.29–2.06 in Model 3) compared with those from the original analysis of Model 3, the fully adjusted Cox regression analysis. (**Table 3 and Table G in S1 File**) Subgroup analysis #1 and #2 also showed similar results in the sensitivity analyses. (**Table 3**)

## Discussion

This study showed that development of ocular motor CN palsy was associated with an increased risk of subsequent stroke, even after adjusting for demographic factors and confounders. Rim et al[6] reported a higher risk for stroke development in patients with ocular motor CN palsy, and the risk to stroke reduced with time only after third and fourth nerve palsies, but not with sixth nerve palsy. They used the same database (NHIS-NSC database) by comparing the risk of stroke between patients with incident CN palsies and their matched controls. However, in the previous study, patients were included for analysis at the time when ocular motor CN palsy occurred. This approach eliminates the period before ocular motor CN palsy in each patient, which is susceptible to selection bias and length time bias. In a longitudinal study using Cox proportional hazard models, development of intermediate events (e.g. ocular motor CN palsy), which can occur at any time of the study period, could substantially affect outcome assessment (e.g. stroke development). To reduce such bias in the present study, we treated the intermediate event–development of ocular motor CN palsy–as a time-varying covariate. In addition, the baseline characteristics were quite different between ocular motor CN palsy and controls in the study by Rim et al; hypertension and diabetes mellitus were more common in ocular motor CN palsy, which decreased the comparability of outcomes. In order to avoid these problems, we carefully designed the three Cox proportional hazards models to adjust for possible risk factors of stroke. We also performed sensitivity analyses by the use of propensity score matching. Therefore, our method can be a more reasonable way to find any change in the incidence of stroke according to the development of ocular motor CN palsy.

In ocular motor CN palsy, the etiology and distribution of affected cranial nerves are variable among reports.[4, 18–20] Among the vast etiologies, microvascular ischemia is by far the most common.[4, 18–20] In our study, the etiology of CN palsy in those with subsequent stroke is presumed to be microvascular ischemic ocular motor CN palsy, as there were no patients with the diagnosis of brain tumor (C69-72, D31-33), viral disease of the CNS (A80-89), multiple sclerosis (G35), and brain aneurysm (I671) within one month from the incident of CN palsy.

Ischemic stroke and ocular motor CN palsy both share common risk factors such as diabetes mellitus, hyperlipidemia, and hypertension.[5] Meanwhile, ocular motor cranial nerves are particularly vulnerable to localized lesions at the level of the brainstem cranial nerve nuclei and the cavernous sinus.[21] Even a silent brain infarction on magnetic resonance imaging with no neurologic symptoms is associated with a 2-fold increased risk of future stroke.[22] Given the fact that not all patients undergo neuroimaging in the setting of presumed microvascular ocular motor nerve palsy, except for third nerve palsy, the possibility of an isolated brainstem infarction to be the cause of cranial nerve palsy may be underestimated. [20] On the other hand, hemorrhagic stroke is seldom the cause of ocular motor CN palsy, and there are only few case reports of isolated ocular motor CN palsy caused by acute brainstem hemorrhage.[23] Accordingly in our study, the risk of hemorrhagic stroke after incident ocular motor CN palsy was not significantly higher compared to controls after adjusting for demographic factors and covariates.

Regarding the type of ocular motor CN palsy, third and sixth nerve palsies were associated with an increased risk of subsequent stroke while fourth nerve palsy was not. Similarly, a population-based study found that the majority of isolated fourth nerve palsies were presumed to be congenital even when presenting in adulthood, and other etiologies such as hypertension and trauma were less frequent.[24]

Compared to the previous studies, this study has the following strong points; firstly, this analysis included the entire population of 20 years or older in a given large population-based database up to 7,008,001 person-years which is the largest ever studied. Secondly, we used time-varying cox proportional hazards regression models to ascertain the effect of ocular motor CN palsy as the variable of interest on stroke occurrence. In addition, in order to control the effect of known risk factors of stroke such as age, sex, socioeconomic status, hypertension, diabetes, ischemic heart disease, atrial fibrillation, congestive heart failure, peripheral artery disease, cancer, tuberculosis, chronic kidney diseases, dyslipidemia, Charlson index scores and co-medications, we applied these factors in the model. [1–3, 25] Thirdly, since these models may not be able to fully control unknown factors in the relationship between ocular motor CN palsy and stroke, we performed a sensitivity analysis by constructing a matched cohort for ocular motor CN palsy based on propensity score matching. As a result, sensitivity analyses showed that the magnitude of the effect of ocular motor CN palsy in COX model 1, 2, and 3 were constant, indicating that the other variables except for ocular motor CN palsy were well addressed between the two groups.

It is quite important to educate patients about modifiable risk factors of stroke. In our study, hypertension, diabetes, ischemic heart disease, congestive heart failure, chronic kidney diseases, peripheral arterial disease, and atrial fibrillation were associated with the development of stroke. Considering the increased risk of stroke after ocular motor CN palsy, adequate patient education should be emphasized on lifestyle modification and search for other risk factors for stroke prevention. It may also be useful to find out whether secondary prevention could reduce the risk of subsequent stroke in such patients. Further investigation is warranted to clarify this issue.

The study has some limitations. Firstly, as a population-based study, diagnostic accuracy in this study depends on the International Classification of Disease codes from the NHIS-NSC database, and the validity of the diagnosis of ocular motor CN palsy has not been evaluated. However, the Korean national health insurance committee has put quite a tremendous effort to enhance the diagnostic accuracy.[10] In addition, the diagnosis of third, fourth and sixth nerve palsy is practically straightforward and the chance of misdiagnosis is relatively low. We excluded all pre-existing conditions of ophthalmoplegia by removing diplopia, other paralytic strabismus, binocular movement disorders, and any other cerebrovascular diseases before the index date. Secondly, there was no information about smoking, alcohol consumption, other metabolic profiles, body mass index, or physical activity in the NHIS-NSC database, which may affect the risk of stroke.

In conclusion, the present results suggest that the incident of ocular motor CN palsy is a significant risk factor of stroke even after adjusting for demographic factors and covariates in the general population. Physicians may need to educate patients about the higher risk of stroke after ocular motor CN palsy for future prevention and life style modification.

## Supporting information

S1 File (Table A) Results of Time-varying Cox Regression Models (Subgroup Analysis #1) for Incident Stroke in the Eligible Population with Third Nerve Palsy as the Time-varying Covariate. (Table B) Results of Time-varying Cox Regression Models (Subgroup Analysis #1) for Incident Stroke in the Eligible Population with Fourth Nerve Palsy as the Time-varying Covariate. (Table C) Results of Time-varying Cox Regression Models (Subgroup Analysis #1) for Incident Stroke in the Eligible Population with Sixth Nerve Palsy as the Time-varying Covariate. (Table D) Results of Time-varying Cox Regression Models (Subgroup Analysis #2) for Ischemic Stroke in the Eligible Population. (Table E) Results of Time-varying Cox Regression Models (Subgroup Analysis #2) for Hemorrhagic Stroke in the Eligible Population. (Table F) Demographics and Characteristics of Patients with Incident Cranial Nerve Palsy and Propensity Score-based Matched Population (1:10) in the Sensitivity Analysis. (Table G) Results of Time-varying Cox Regression Models for Incident Stroke in the Propensity Score-based Sensitivity Analysis.
(DOCX)
