# Supplementary material for: Ocular motor cranial nerve palsy and increased risk of stroke in the general population
Source: PLoS One. 2018 Oct 15;13(10):e0205428. doi: 10.1371/journal.pone.0205428 (PMC6188901; doi:10.1371/journal.pone.0205428)
Supplement: S1 File — (Table A) Results of Time-varying Cox Regression Models (Subgroup Analysis #1) for Incident Stroke in the Eligible Population with Third Nerve Palsy as the Time-varying Covariate. (Table B) Results of Time-varying Cox Regression Models (Subgroup Analysis #1) for Incident Stroke in the Eligible Population with Fourth Nerve Palsy as the Time-varying Covariate. (Table C) Results of Time-varying Cox Regression Models (Subgroup Analysis #1) for Incident Stroke in the Eligible Population with Sixth Nerve Palsy as the Time-varying Covariate. (Table D) Results of Time-varying Cox Regression Models (Subgroup Analysis #2) for Ischemic Stroke in the Eligible Population. (Table E) Results of Time-varying Cox Regression Models (Subgroup Analysis #2) for Hemorrhagic Stroke in the Eligible Population. (Table F) Demographics and Characteristics of Patients with Incident Cranial Nerve Palsy and Propensity Score-based Matched Population (1:10) in the Sensitivity Analysis. (Table G) Results of Time-varying Cox Regression Models for Incident Stroke in the Propensity Score-based Sensitivity Analysis. (DOCX) [file pone.0205428.s001.docx]

**Table A. Results of Time-varying Cox Regression Models (Subgroup Analysis #1) for Incident Stroke in the Eligible Population with Third Nerve Palsy as the Time-varying Covariate.**

|  | Model 1 | Model 2 | Model 3 |
| --- | --- | --- | --- |
| **Time-varying Covariate** |  |  |  |
| Third Nerve Palsy | 5.65 (3.46-9.23) | 2.67 (1.63-4.36) | 2.15 (1.32-3.51) |
|  |  |  |  |
| **Demographics** |  |  |  |
| Sex |  |  |  |
| Men | N/A | 1 (reference) | 1 (reference) |
| Women | N/A | 0.73 (0.71-0.75) | 0.72 (0.70-0.74) |
| Age Group (years) |  |  |  |
| 20-39 | N/A | 1 (reference) | 1 (reference) |
| 40-59 | N/A | 6.02 (5.65-6.41) | 4.26 (3.99-4.55) |
| 60+ | N/A | 31.92 (30.05-33.91) | 15.11 (14.16-16.14) |
| Residence |  |  |  |
| Seoul and Incheon | N/A | 1 (reference) | 1 (reference) |
| Gyeonggi and Gangwon | N/A | 1.15 (1.10-1.21) | 1.14 (1.09-1.20) |
| Busan, Daegu, Ulsan, and Gyeongsang | N/A | 1.32 (1.26-1.37) | 1.37 (1.32-1.43) |
| Daejeon, Sejong, and Chungcheong | N/A | 1.25 (1.19-1.32) | 1.26 (1.19-1.33) |
| Gwangju, Jeola, and Jeju | N/A | 1.25 (1.19-1.31) | 1.28 (1.21-1.34) |
| House Income |  |  |  |
| Low income | N/A | 1 (reference) | 1 (reference) |
| Middle income | N/A | 0.92 (0.89-0.96) | 0.89 (0.86-0.92) |
| High income | N/A | 0.88 (0.85-0.91) | 0.83 (0.80-0.86) |
|  |  |  |  |
| **Comorbidity*** |  |  |  |
| Hypertension | N/A | N/A | 2.53 (2.43-2.63) |
| Diabetes | N/A | N/A | 1.29 (1.24-1.33) |
| Ischemic Heart Disease | N/A | N/A | 1.14 (1.10-1.19) |
| Congestive Heart Failure | N/A | N/A | 1.34 (1.28-1.40) |
| Cancer | N/A | N/A | 1.00 (0.96-1.05) |
| Tuberculosis | N/A | N/A | 1.03 (0.97-1.10) |
| Peripheral Arterial Disease | N/A | N/A | 1.15 (1.11-1.19) |
| Atrial fibrillation | N/A | N/A | 2.31 (2.05-2.60) |
| Chronic Kidney Disease | N/A | N/A | 1.57 (1.43-1.72) |
| Dyslipidemia | N/A | N/A | 0.93 (0.90-0.96) |
|  |  |  |  |
| **Co-medications** |  |  |  |
| Anti-coagulant agents | N/A | N/A | 1.43 (1.14-1.78) |
| Anti-hypertensive agents | N/A | N/A | 0.91 (0.87-0.95) |
| Anti-platelet agents | N/A | N/A | 1.05 (0.95-1.16) |
| Hypoglycemic agents | N/A | N/A | 2.61 (0.84-8.08) |

* Comorbidities were treated as time-varying covariates; Model 3 included Charlson comorbidity index in the analysis; N/A, not applicable

**Table B. Results of Time-varying Cox Regression Models (Subgroup Analysis #1) for Incident Stroke in the Eligible Population with Fourth Nerve Palsy as the Time-varying Covariate.**

|  | Model 1 | Model 2 | Model 3 |
| --- | --- | --- | --- |
| **Time-varying Covariate** |  |  |  |
| Fourth Nerve Palsy | 3.93 (2.04-7.55) | 1.99 (1.04-3.83) | 1.72 (0.90-3.31) |
|  |  |  |  |
| **Demographics** |  |  |  |
| Sex |  |  |  |
| Men | N/A | 1 (reference) | 1 (reference) |
| Women | N/A | 0.73 (0.71-0.75) | 0.72 (0.70-0.74) |
| Age Group (years) |  |  |  |
| 20-39 | N/A | 1 (reference) | 1 (reference) |
| 40-59 | N/A | 6.02 (5.65-6.41) | 4.26 (3.99-4.55) |
| 60+ | N/A | 31.93 (30.06-33.92) | 15.11 (14.16-16.14) |
| Residence |  |  |  |
| Seoul and Incheon | N/A | 1 (reference) | 1 (reference) |
| Gyeonggi and Gangwon | N/A | 1.15 (1.10-1.21) | 1.14 (1.09-1.19) |
| Busan, Daegu, Ulsan, and Gyeongsang | N/A | 1.32 (1.26-1.38) | 1.38 (1.32-1.44) |
| Daejeon, Sejong, and Chungcheong | N/A | 1.25 (1.19-1.32) | 1.26 (1.19-1.33) |
| Gwangju, Jeola, and Jeju | N/A | 1.25 (1.19-1.31) | 1.27 (1.21-1.34) |
| House Income |  |  |  |
| Low income | N/A | 1 (reference) | 1 (reference) |
| Middle income | N/A | 0.92 (0.89-0.96) | 0.89 (0.86-0.92) |
| High income | N/A | 0.88 (0.85-0.91) | 0.83 (0.80-0.86) |
|  |  |  |  |
| **Comorbidity*** |  |  |  |
| Hypertension | N/A | N/A | 2.52 (2.42-2.62) |
| Diabetes | N/A | N/A | 1.29 (1.24-1.33) |
| Ischemic Heart Disease | N/A | N/A | 1.11 (1.07-1.16) |
| Congestive Heart Failure | N/A | N/A | 1.29 (1.23-1.35) |
| Cancer | N/A | N/A | 1.00 (0.95-1.04) |
| Tuberculosis | N/A | N/A | 1.02 (0.95-1.08) |
| Peripheral Arterial Disease | N/A | N/A | 1.15 (1.11-1.20) |
| Atrial fibrillation | N/A | N/A | 1.79 (1.68-1.91) |
| Chronic Kidney Disease | N/A | N/A | 1.56 (1.42-1.71) |
| Dyslipidemia | N/A | N/A | 0.93 (0.89-0.96) |
|  |  |  |  |
| **Co-medications** |  |  |  |
| Anti-coagulant agents | N/A | N/A | 1.11 (0.89-1.40) |
| Anti-hypertensive agents | N/A | N/A | 0.91 (0.87-0.95) |
| Anti-platelet agents | N/A | N/A | 1.03 (0.93-1.13) |
| Hypoglycemic agents | N/A | N/A | 2.69 (0.87-8.33) |

* Comorbidities were treated as time-varying covariates; Model 3 included Charlson comorbidity index in the analysis; N/A, not applicable

**Table C. Results of Time-varying Cox Regression Models (Subgroup Analysis #1) for Incident Stroke in the Eligible Population with Sixth Nerve Palsy as the Time-varying Covariate.**

|  | Model 1 | Model 2 | Model 3 |
| --- | --- | --- | --- |
| **Time-varying Covariate** |  |  |  |
| Sixth Nerve Palsy | 4.58 (2.76-7.61) | 2.40 (1.44-3.98) | 1.83 (1.10-3.04) |
|  |  |  |  |
| **Demographics** |  |  |  |
| Sex |  |  |  |
| Men | N/A | 1 (reference) | 1 (reference) |
| Women | N/A | 0.73 (0.71-0.75) | 0.72 (0.70-0.74) |
| Age Group (years) |  |  |  |
| 20-39 | N/A | 1 (reference) | 1 (reference) |
| 40-59 | N/A | 6.02 (5.65-6.41) | 4.26 (3.99-4.55) |
| 60+ | N/A | 31.92 (30.05-33.91) | 15.12 (14.16-16.14) |
| Residence |  |  |  |
| Seoul and Incheon | N/A | 1 (reference) | 1 (reference) |
| Gyeonggi and Gangwon | N/A | 1.15 (1.10-1.21) | 1.14 (1.09-1.19) |
| Busan, Daegu, Ulsan, and Gyeongsang | N/A | 1.32 (1.26-1.37) | 1.38 (1.32-1.44) |
| Daejeon, Sejong, and Chungcheong | N/A | 1.25 (1.19-1.32) | 1.26 (1.19-1.33) |
| Gwangju, Jeola, and Jeju | N/A | 1.25 (1.19-1.31) | 1.27 (1.21-1.34) |
| House Income |  |  |  |
| Low income | N/A | 1 (reference) | 1 (reference) |
| Middle income | N/A | 0.92 (0.89-0.96) | 0.89 (0.86-0.92) |
| High income | N/A | 0.88 (0.85-0.91) | 0.83 (0.80-0.86) |
|  |  |  |  |
| **Comorbidity*** |  |  |  |
| Hypertension | N/A | N/A | 2.52 (2.42-2.62) |
| Diabetes | N/A | N/A | 1.29 (1.24-1.33) |
| Ischemic Heart Disease | N/A | N/A | 1.11 (1.07-1.16) |
| Congestive Heart Failure | N/A | N/A | 1.29 (1.23-1.35) |
| Cancer | N/A | N/A | 1.00 (0.95-1.04) |
| Tuberculosis | N/A | N/A | 1.02 (0.95-1.08) |
| Peripheral Arterial Disease | N/A | N/A | 1.15 (1.11-1.20) |
| Atrial fibrillation | N/A | N/A | 1.79 (1.68-1.91) |
| Chronic Kidney Disease | N/A | N/A | 1.56 (1.42-1.71) |
| Dyslipidemia | N/A | N/A | 0.93 (0.89-0.96) |
|  |  |  |  |
| **Co-medications** |  |  |  |
| Anti-coagulant agents | N/A | N/A | 1.11 (0.89-1.39) |
| Anti-hypertensive agents | N/A | N/A | 0.91 (0.87-0.95) |
| Anti-platelet agents | N/A | N/A | 1.03 (0.93-1.13) |
| Hypoglycemic agents | N/A | N/A | 2.69 (0.87-8.34) |

* Comorbidities were treated as time-varying covariates; Model 3 included Charlson comorbidity index in the analysis; N/A, not applicable

**Table D. Results of Time-varying Cox Regression Models (Subgroup Analysis #2) for Ischemic Stroke in the Eligible Population.**

|  | Model 1 | Model 2 | Model 3 |
| --- | --- | --- | --- |
| **Time-varying Covariate** |  |  |  |
| Cranial Nerve Palsy | 5.31 (4.20-6.72) | 2.41 (1.90-3.05) | 1.81 (1.43-2.29) |
|  |  |  |  |
| **Demographics** |  |  |  |
| Sex |  |  |  |
| Men | N/A | 1 (reference) | 1 (reference) |
| Women | N/A | 0.72 (0.69-0.74) | 0.69 (0.67-0.72) |
| Age Group (years) |  |  |  |
| 20-39 | N/A | 1 (reference) | 1 (reference) |
| 40-59 | N/A | 8.57 (7.84-9.38) | 5.74 (5.24-6.29) |
| 60+ | N/A | 55.24 (50.67-60.22) | 23.83 (21.75-26.12) |
| Residence |  |  |  |
| Seoul and Incheon | N/A | 1 (reference) | 1 (reference) |
| Gyeonggi and Gangwon | N/A | 1.18 (1.12-1.25) | 1.17 (1.11-1.23) |
| Busan, Daegu, Ulsan, and Gyeongsang | N/A | 1.40 (1.33-1.47) | 1.47 (1.40-1.55) |
| Daejeon, Sejong, and Chungcheong | N/A | 1.33 (1.25-1.41) | 1.34 (1.26-1.43) |
| Gwangju, Jeola, and Jeju | N/A | 1.31 (1.23-1.39) | 1.34 (1.27-1.43) |
| House Income |  |  |  |
| Low income | N/A | 1 (reference) | 1 (reference) |
| Middle income | N/A | 0.93 (0.89-0.97) | 0.89 (0.85-0.92) |
| High income | N/A | 0.91 (0.87-0.95) | 0.84 (0.81-0.88) |
|  |  |  |  |
| **Comorbidity*** |  |  |  |
| Hypertension | N/A | N/A | 2.66 (2.54-2.79) |
| Diabetes | N/A | N/A | 1.39 (1.33-1.44) |
| Ischemic Heart Disease | N/A | N/A | 1.13 (1.08-1.18) |
| Congestive Heart Failure | N/A | N/A | 1.32 (1.25-1.39) |
| Cancer | N/A | N/A | 0.92 (0.88-0.97) |
| Tuberculosis | N/A | N/A | 1.03 (0.96-1.10) |
| Peripheral Arterial Disease | N/A | N/A | 1.18 (1.13-1.23) |
| Atrial fibrillation | N/A | N/A | 1.89 (1.76-2.02) |
| Chronic Kidney Disease | N/A | N/A | 1.40 (1.26-1.56) |
| Dyslipidemia | N/A | N/A | 0.97 (0.93-1.01) |
|  |  |  |  |
| **Co-medications** |  |  |  |
| Anti-coagulant agents | N/A | N/A | 0.92 (0.71-1.20) |
| Anti-hypertensive agents | N/A | N/A | 0.92 (0.88-0.97) |
| Anti-platelet agents | N/A | N/A | 0.98 (0.88-1.10) |
| Hypoglycemic agents | N/A | N/A | 3.04 (0.98-9.44) |

* Comorbidities were treated as time-varying covariates; Model 3 included Charlson comorbidity index in the analysis; N/A, not applicable

**Table E. Results of Time-varying Cox Regression Models (Subgroup Analysis #2) for Hemorrhagic Stroke in the Eligible Population.**

|  | Model 1 | Model 2 | Model 3 |
| --- | --- | --- | --- |
| **Time-varying Covariate** |  |  |  |
| Cranial Nerve Palsy | 2.78 (1.64-4.69) | 1.53 (0.90-2.58) | 1.22 (0.72-2.06) |
|  |  |  |  |
| **Demographics** |  |  |  |
| Sex |  |  |  |
| Men | N/A | 1 (reference) | 1 (reference) |
| Women | N/A | 0.77 (0.73-0.81) | 0.79 (0.74-0.83) |
| Age Group (years) |  |  |  |
| 20-39 | N/A | 1 (reference) | 1 (reference) |
| 40-59 | N/A | 4.01 (3.67-4.39) | 3.10 (2.82-3.40) |
| 60+ | N/A | 12.04 (11.02-13.15) | 6.71 (6.07-7.41) |
| Residence |  |  |  |
| Seoul and Incheon | N/A | 1 (reference) | 1 (reference) |
| Gyeonggi and Gangwon | N/A | 1.07 (0.98-1.16) | 1.06 (0.97-1.15) |
| Busan, Daegu, Ulsan, and Gyeongsang | N/A | 1.17 (1.08-1.26) | 1.20 (1.11-1.30) |
| Daejeon, Sejong, and Chungcheong | N/A | 1.11 (1.00-1.23) | 1.10 (0.99-1.22) |
| Gwangju, Jeola, and Jeju | N/A | 1.12 (1.01-1.23) | 1.13 (1.03-1.25) |
| House Income |  |  |  |
| Low income | N/A | 1 (reference) | 1 (reference) |
| Middle income | N/A | 0.92 (0.86-0.98) | 0.91 (0.85-0.97) |
| High income | N/A | 0.78 (0.73-0.84) | 0.76 (0.71-0.82) |
|  |  |  |  |
| **Comorbidity*** |  |  |  |
| Hypertension | N/A | N/A | 2.50 (2.33-2.69) |
| Diabetes | N/A | N/A | 1.08 (1.01-1.16) |
| Ischemic Heart Disease | N/A | N/A | 1.03 (0.95-1.12) |
| Congestive Heart Failure | N/A | N/A | 1.12 (1.02-1.24) |
| Cancer | N/A | N/A | 1.23 (1.13-1.35) |
| Tuberculosis | N/A | N/A | 1.02 (0.90-1.16) |
| Peripheral Arterial Disease | N/A | N/A | 1.00 (0.92-1.07) |
| Atrial fibrillation | N/A | N/A | 1.96 (1.72-2.22) |
| Chronic Kidney Disease | N/A | N/A | 2.12 (1.79-2.51) |
| Dyslipidemia | N/A | N/A | 0.85 (0.79-0.91) |
|  |  |  |  |
| **Co-medications** |  |  |  |
| Anti-coagulant agents | N/A | N/A | 2.04 (1.40-2.97) |
| Anti-hypertensive agents | N/A | N/A | 0.78 (0.71-0.86) |
| Anti-platelet agents | N/A | N/A | 1.18 (0.96-1.46) |
| Hypoglycemic agents | N/A | N/A | 0 |

* Comorbidities were treated as time-varying covariates; Model 3 included Charlson comorbidity index in the analysis; N/A, not applicable

**Table F. Demographics** **and Characteristics of Patients with Incident Cranial Nerve Palsy and Propensity Score-based Matched Population (1:10) in the Sensitivity Analysis.**

|  | **Patients with**  **Cranial Nerve Palsy** | **Matched Population without**  **Cranial Nerve Palsy** | **SMD** |
| --- | --- | --- | --- |
| Number | 1,633 (100%) | 16,330 (100%) |  |
|  |  |  |  |
| **Demographics** |  |  |  |
| Sex |  |  |  |
| Women | 728 (44.6%) | 7,229 (44.3%) | 0.63 |
| Men | 905 (55.4%) | 9,101 (55.7%) | -0.36 |
| Age group at diagnosis (years) |  |  |  |
| 20-39 | 232 (14.2%) | 2,308 (14.1%) | 0.21 |
| 40-59 | 765 (46.8%) | 7,359 (45.1%) | 3.58 |
| 60+ | 636 (38.9%) | 6,663 (40.8%) | -3.79 |
| Residential Area |  |  |  |
| Seoul and Incheon | 430 (26.3%) | 4,316 (26.4%) | -0.22 |
| Gyeonggi and Gangwon | 372 (22.8%) | 3,731 (22.8%) | -0.16 |
| Busan, Daegu, Ulsan, and Gyeongsang | 442 (27.1%) | 4,455 (27.3%) | -0.48 |
| Daejeon, Sejong, and Chungcheong | 176 (10.8%) | 1,675 (10.3%) | 1.70 |
| Gwangju, Jeola, and Jeju | 213 (13.0%) | 2,153 (13.2%) | -0.42 |
| House Income |  |  |  |
| Low income | 524 (32.1%) | 5,150 (31.5%) | 1.18 |
| Middle income | 631 (38.6%) | 6,412 (39.3%) | -1.28 |
| High income | 478 (29.3%) | 4,768 (29.2%) | 0.16 |
|  |  |  |  |
| **Comorbidity** |  |  |  |
| Hypertension | 505 (30.9%) | 5,027 (30.8%) | 0.74 |
| Diabetes | 490 (30.0%) | 4,826 (29.6%) | 0.84 |
| Ischemic Heart Disease | 144 (8.8%) | 1,466 (9.0%) | 0.70 |
| Congestive Heart Failure | 52 (3.2%) | 499 (3.1%) | 0.59 |
| Cancer | 76 (4.7%) | 670 (4.1%) | 0.60 |
| Tuberculosis | 26 (1.6%) | 267 (1.6%) | 0.34 |
| Peripheral Arterial Disease | 80 (4.9%) | 781 (4.8%) | 0.67 |
| Atrial Fibrillation | 17 (1.0%) | 151 (0.9%) | 0.77 |
| Chronic Kidney Disease | 13 (0.8%) | 131 (0.8%) | -0.07 |
| Dyslipidemia | 304 (18.6%) | 2,977 (18.2%) | 1.00 |
|  |  |  |  |
| **Co-medication** |  |  |  |
| Anti-coagulant agents | 7 (0.4%) | 66 (0.4%) | 0.38 |
| Anti-hypertensive agents | 244 (14.9%) | 2,469 (15.1%) | -0.50 |
| Anti-platelet agents | 37 (2.3%) | 377 (2.3%) | -0.29 |
| Hypoglycemic agents | 1 (0.1%) | 10 (0.1%) | 0.00 |

SMD, standardized mean differences**Table G. Results of Time-varying Cox Regression Models for Incident Stroke in the Propensity Score-based Sensitivity Analysis.**

|  | Model 1 | Model 2 | Model 3 |
| --- | --- | --- | --- |
| **Time-varying Covariate** |  |  |  |
| Cranial Nerve Palsy | 1.95 (1.55-2.46) | 1.91 (1.52-2.41) | 1.63 (1.29-2.06) |
|  |  |  |  |
| **Demographics** |  |  |  |
| Sex |  |  |  |
| Men | N/A | 1 (reference) | 1 (reference) |
| Women | N/A | 0.76 (0.67-0.86) | 0.75 (0.66-0.85) |
| Age Group (years) |  |  |  |
| 20-49 | N/A | 1 (reference) | 1 (reference) |
| 40-59 | N/A | 4.00 (2.54-6.30) | 2.70 (1.70-4.28) |
| 60+ | N/A | 15.35 (9.84-23.95) | 7.36 (4.65-11.64) |
| Residence |  |  |  |
| Seoul and Incheon | N/A | 1 (reference) | 1 (reference) |
| Gyeonggi and Gangwon | N/A | 0.99 (0.83-1.20) | 0.98 (0.81-1.18) |
| Busan, Daegu, Ulsan, and Gyeongsang | N/A | 1.25 (1.06-1.48) | 1.32 (1.12-1.57) |
| Daejeon, Sejong, and Chungcheong | N/A | 1.29 (1.03-1.60) | 1.32 (1.06-1.64) |
| Gwangju, Jeola, and Jeju | N/A | 1.10 (0.90-1.36) | 1.21 (0.98-1.49) |
| House Income |  |  |  |
| Low income | N/A | 1 (reference) | 1 (reference) |
| Middle income | N/A | 0.98 (0.84-1.14) | 0.95 (0.82-1.11) |
| High income | N/A | 1.14 (0.98-1.33) | 1.10 (0.94-1.28) |
|  |  |  |  |
| **Comorbidity*** |  |  |  |
| Hypertension | N/A | N/A | 2.32 (1.95-2.78) |
| Diabetes | N/A | N/A | 1.34 (1.15-1.55) |
| Ischemic Heart Disease | N/A | N/A | 1.09 (0.94-1.26) |
| Congestive Heart Failure | N/A | N/A | 1.22 (1.03-1.46) |
| Cancer | N/A | N/A | 0.88 (0.73-1.07) |
| Tuberculosis | N/A | N/A | 1.04 (0.81-1.33) |
| Peripheral Arterial Disease | N/A | N/A | 1.19 (1.04-1.37) |
| Atrial fibrillation | N/A | N/A | 1.95 (1.54-2.47) |
| Chronic Kidney Disease | N/A | N/A | 1.76 (1.33-2.32) |
| Dyslipidemia | N/A | N/A | 0.88 (0.76-1.02) |
|  |  |  |  |
| **Co-medications** |  |  |  |
| Anti-coagulant agents | N/A | N/A | 0.90 (0.49-1.64) |
| Anti-hypertensive agents | N/A | N/A | 0.94 (0.81-1.11) |
| Anti-platelet agents | N/A | N/A | 1.08 (0.80-1.47) |
| Hypoglycemic agents | N/A | N/A | 2.41 (0.60-9.71) |

* Comorbidities were treated as time-varying covariates; Model 3 included Charlson comorbidity index in the analysis; N/A, not applicable
